# Effects of High Altitude on Sleep and Respiratory System and Theirs Adaptations

**DOI:** 10.1155/2013/241569

**Published:** 2013-04-17

**Authors:** Turhan San, Senol Polat, Cemal Cingi, Gorkem Eskiizmir, Fatih Oghan, Burak Cakir

**Affiliations:** ^1^Department of Otolaryngology-Head and Neck Surgery, Istanbul Medeniyet University, Faculty of Medicine, 34100 Istanbul, Turkey; ^2^Department of Otolaryngology-Head and Neck Surgery, Acibadem University, Faculty of Medicine, 34742 Istanbul, Turkey; ^3^Department of Otolaryngology-Head and Neck Surgery, Osmangazi University, Faculty of Medicine, 26020 Eskisehir, Turkey; ^4^Department of Otolaryngology-Head and Neck Surgery, Celal Bayar University, Faculty of Medicine, 45010 Manisa, Turkey; ^5^Department of Otolaryngology-Head and Neck Surgery, Dumlupinar University, Faculty of Medicine, 43100 Kutahya, Turkey; ^6^Sisli Etfal Training and Research Hospital, Department of Otolaryngology-Head and Neck Surgery, 34371 Istanbul, Turkey

## Abstract

High-altitude (HA) environments have adverse effects on the normal functioning body of people accustomed to living at low altitudes because of the change in barometric pressure which causes decrease in the amount of oxygen leading to hypobaric hypoxia. Sustained exposure to hypoxia has adverse effects on body weight, muscle structure and exercise capacity, mental functioning, and sleep quality. The most important step of acclimatization is the hyperventilation which is achieved by hypoxic ventilatory response of the peripheral chemoreceptors. Hyperventilation results in increase in arterial carbondioxide concentration. Altitude also affects sleep and cardiac output, which is the other determinant of oxygen delivery. Upon initial exposure to HA, the resting pulse rate increases rapidly, but with acclimatization, heart rate and cardiac output tend to fall. Another important component that leads to decrease in cardiac output is the reduction in the stroke volume with acclimatization. During sleep at HA, the levels of CO_2_ in the blood can drop very low and this can switch off the drive to breathe. Only after the body senses a further drop in O_2_ levels breathing is started again. Periodic breathing is thought to result from instability in the control system through the hypoxic drive or the response to CO_2_.

## 1. Introduction

Modern travel facilities and mountain tours now permit access to high mountains previously visited only rarely by hardy climbers. Traveling to elevations over 2500 meters may lead to signs and symptoms of HA illness [1]. Effects of high-altitude (HA) depend on several factors, including the rate of ascent to altitude, final altitude reached, altitude at which a person sleeps, and individual physiology [2–4].

On arriving at HA, lowlanders will be incapable of as much physical exertion as they were at sea level. Further, they may not feel well and may have impaired mentation. These effects are ultimately due to hypoxia. Fortunately, human body has a series of physiological adjustments to compensate this hypoxia including increase in ventilation, hemodynamic and hematologic changes, and metabolic changes which are usually termed as acclimatization [2, 3].

The time required for these adaptations varies with the individual physiology, with the altitude ascended and with the speed of ascent [4]. This paper reviews the effects of HA, as well as the adaptations to the changes associated with HA.

## 2. Oxygen at HA

HA that reflects the lowered amount of gases including O_2_ in the atmosphere is defined as [5]:

- intermediate altitude: 1500–2500 m;
- HA: 2500–3500 m;
- very HA: 3500–5800 m;
- extreme altitude: above 5800 m.

Air is a mixture of gases and the principal gases are O_2_ and nitrogen whose summated partial pressures equal the barometric pressure (BP). Their concentrations are essentially constant over earth terrestrial elevations [3]. Thus, the amount of O_2_ in the atmosphere, 20.93 percent, remains constant at any given altitude. However, the surface of earth oceans, which we call sea level, is also the bottom of an ocean of air and air, unlike water, is compressible.

The partial pressure of O_2_ (PaO_2_) in the atmosphere falls as BP falls. Therefore, the change in BP at HA is the basic cause of decrease in the amount of O_2_ leading to hypobaric hypoxia (HH) [6, 7]. Atmospheric pressure and the PaO_2_ decrease at increasing altitude in a logarithmic fashion. The atmospheric PaO_2_ is 159 mm Hg at sea level and 53 mm Hg on the summit of Mount Everest [8, 9]. Although the major determining factor of PaO_2_ is BP, the PaO_2_ is also lowered towards the poles of the earth at any given altitude. It should also be noted that BP is known to fluctuate with changing weather systems [2].

## 3. Effects of HA

When the climbers are exposed to HH, they experienced different reactions to the effects of altitude. The basis of pathophysiological changes is tissue HH. The greater the hypoxic stress, the less time the body has to adapt to it and the greater the adverse effects of HA.

## 4. Maximal Oxygen Consumption (VO_2 max_)

VO_2 max⁡_ is the maximum capacity of an individual's body to transport and use O_2_ during exercise, which reflects the physical fitness of the individual. The point at which O_2_ consumption plateaus defines the individual's maximal aerobic capacity. This capacity varies among the individuals and can be improved to a level with training. Genetics plays a major role in a person's VO_2 max⁡_, and heredity can account for up to 25–50% of the variance seen between individuals [6].

VO_2 max⁡_ begins to decrease significantly above an altitude of 1600 m. For every 1000 m above that VO_2 max⁡  _ drops by approximately 8–11%. At the summit of Everest, an average sea level VO_2  max⁡_ of 62 mL/kg/min can drop to 15 mL/kg/min. Anyone with a VO_2 max⁡_ lower than 50 mL/kg/min would struggle to survive at the summit of Everest without supplemental O_2_ [7].

Since at altitude the transfer of O_2_ to the active muscles is reduced, particularly during whole body exercise, fatigue occurs at lower work rates [4, 8]. The reduced VO_2 max⁡_ at HA is usually ascribed to the reduction in mitochondrial PO_2_, which interferes with the function of the electron transport chain responsible for providing cellular energy [3, 8]. Although arterial O_2_ content increases to values of sea level with acclimatization VO_2 max⁡_ capacity remains reduced [3, 9]. The reason was proposed to be the unproportionate delivery of O_2_ to the tissues; while under sea level conditions O_2_ is more directed to contracting muscles during exercise, at HAs greater proportion of the O_2_ is directed to noncontracting tissues during exercise. Thus exercise performance is reduced. There is little evidence that the pulmonary hypertension of HA limits VO_2 max⁡_ [10].

## 5. Skeletal Muscle and Body Weight

Sustained exposure to severe hypoxia has detrimental effects on muscle structure. Chronic hypoxia of altitude leads to a marked decrease in muscle fiber density [4, 11]. Similarly, there is a decrease in mitochondrial volume by up to 30% [12]. The changes in mitochondrial volume are accompanied by significant decrease in the activity of enzymes responsible for aerobic oxidative metabolism and muscle oxidative capacity and are found to be moderately reduced by exposure to altitude. In contrast, proteins involved in the cellular transport of bicarbonate, protons, and lactates are increased in both skeletal muscle and red blood cells (RBCs) [13, 14]. Prolonged exposure to HH which causes reduction in maximal rate of O_2_ uptake was proposed as the main reason for decrease in muscle cross-sectional area and in muscle oxidative activity [12]. These changes correlate with body weight and overall muscle mass decline at HA.

Hypoxia also causes diaphragm and abdominal muscle contractile fatigue which results in exercise performance limitations at HAs [15]. Despite these negative effects of HH, Edwards et al. [16] suggested that in response to HA hypoxia, skeletal muscle function is maintained in humans, although there is a significant muscle atrophy. The proposed causes of weight loss (WL) at HA are decreased food intake due to loss of appetite, changes in endocrine parameters controlling homeostasis, imbalance of energy intake and expenditure, increased basal metabolic rate and high activity levels, impaired intestinal function, change in body composition including loss of fat mass or loss of muscle mass, and decrease in body water [17–19]. Although it was suggested that body water was gradually lost through increased ventilation and decreased water intake or altered metabolism, some other studies concluded that water balance is maintained at altitude, via increased intake or metabolic water formation, and does not account for WL [17, 18, 20]. From these suggested mechanisms for WL at HAs it is obvious that there is a negative energy balance due to a combination of decreased energy intake and an increased energy expenditure [21]. Loss of appetite with altitude was thought be due to increased leptin levels which is a protein hormone secreted from adipose tissue in response to food intake and affects the satiety center of hypothalamus [17, 18]. However, some studies could not be able to prove this increase and even some other studies showed a decrease in the level of leptin levels at HAs [22]. Changes in body composition result from hypoxia-related suppression of muscle protein synthesis results in WL [17, 20]. Hypoxia-induced intestinal dysfunction contributes to WL especially above 5500 m, and the reason was suggested to be changes in the intestinal flora due to hypoxia [17, 23].

During the study period of the Operation Everest (OE)-II project, weight was found to be reduced by 7.44 kg, which constituted overall 8.9% decline in the body weight [4, 24]. In the same study, in 6 subjects, total muscle area of the thigh and upper arms was calculated via CT scans and the results showed decrease of 13% and 15%, respectively. Weight was reduced by an average of 5 kg in the study participants during the study period of the OE-III which evaluated the long-term effect of HH on appetite using a hypobaric chamber and simulating the ascent of Everest during a 31-day period [25]. They concluded that exposure to HH appeared to be associated with a change in the attitude towards eating and with a decreased appetite and food intake.

## 6. Mental Performance (MP)

HAs of more than 3.000 m produce physiological disorders and adverse changes in moods and cognitive/motor performance of nonacclimatized individuals [3]. It is known that exposure to HA can produce adverse effects in motor skills, mental efficiency, and mood states, including anxiety depending on the altitude level reached, the speed of the ascent, and the time spent at HA [26, 27]. Most people working at an altitude of 4000 m experience an increased number of arithmetic errors, reduced attention span, and increased mental fatigue. Visual and auditory sensitiveness and short-term memory are negatively affected by exposure to an altitude of nearly 2500 m.

The molecular and cellular mechanisms responsible for impaired MP during hypoxia are poorly understood. The brain normally accounts for approximately 20% of the body's total O_2_ consumption, and the O_2_ is almost entirely used for the oxidation of glucose. Suggested mechanisms for the impairment of nerve cell function during hypoxia include altered ion homeostasis, changes in calcium metabolism, alterations in neurotransmitter metabolism, and impairment of synapse function [3, 26, 27]. Cardiovascular and respiratory functions also affect MP and may cause a condition like organic brain syndrome during climbing to HA [28]. Environmental factors, air condition, exercise, and individual differences during climbing to altitude also can have some negative effects on MP [3, 28].

## 7. Sleep

At altitude, the reduced oxygen content of the blood induces breathing instability, with periods of deep and rapid breathing alternating with central apnea. This breathing pattern is called high-altitude periodic breathing (PB). It occurs even in healthy persons at altitudes above 6000 ft. It may lead to sleep disturbances with frequent awakenings and a feeling of lack of air [29]. De Aquino Lemos et al. found that hypoxia reduced total sleep time, sleep efficiency, slow-wave sleep, and rapid eye movement. Depressive mood, anger, and fatigue increased under hypoxic conditions. Vigor, attention, visual and working memory, concentration, executive functions, inhibitory control, and speed of mental processing worsened. Changes in sleep patterns can modulate mood and cognition after 24 h [30]. People at HA often wake frequently, have arousals, and do not feel refreshed in the morning and during day, and they experience somnolence [31]. The periodic breathing (PB) that occurs in most of the people at altitudes above 4000 m is probably the main causative factor [32, 33]. Latshang et al. described that at high altitude, nocturnal periodic breathing affects males more than females. In this study, females started to present a significant number of central sleep apneas only at the highest reached altitude. After 10 days at 5400 m gender differences in the apnea-hypopnea index similar to those observed after acute exposure were still observed, accompanied by differences in respiratory cycle length [34].

PB involves alternating periods of deep breathing and shallow breathing. Typically, three to five deep breaths will be followed by a couple of very shallow breaths or even a complete pause in breathing which is called apnea [32]. During sleep at HA, the levels of CO_2_ in the blood can drop very low and this can switch off the drive to breathe. Only after the body senses a further drop in O_2_ levels breathing is started again. PB is thought to result from instability in the control system through the hypoxic drive or the response to CO_2_ [31, 32]. Weil [31] stated that the sleep disorder of altitude was largely due to respiratory disturbance arising from the physiologic ventilatory dilemma of acute ascent, where stimulation by hypoxia alternates with inhibition by hypocapnic alkalosis. The OE-II decompression chamber studies found severe sleep fragmentation and PB at all altitudes was studied but especially at the HAs. These brief 2- to 5-second arousals from sleep increased from an average of 22 times per hour at sea level to 161 times per hour at 25,000 ft [35, 36]. Although total sleep time was reported to not change, it was found that there was a strong shift from deeper to lighter sleep stages and a marked increase in frequency of brief arousals [37]. Experienced trekkers and mountain climbers often recommend climbing high but sleeping mitigates these problems. The cold, the wind, noisy, or smelly tent companions and long distance travel can also disturb the sleep. Nussbaumer-Ochsner et al. concluded that in healthy mountaineers ascending rapidly to high altitude, sleep quality is initially impaired but improves with acclimatization in association with improved oxygen saturation, while periodic breathing persists. Therefore, high-altitude sleep disturbances seem to be related predominantly to hypoxemia rather than to periodic breathing [38].

## 8. Acclimatization

### 8.1. Oxygen Transport

O_2_ must continuously be transported from the air to the mitochondria in sufficient quantities in order to meet tissue demands. Because the O_2_ amount falls sequentially and progressively, transport can be regarded as a series of steps in a cascade from alveolus to the cells mitochondria [9].

Because the atmospheric PaO_2_ is lower at HAs, gradient driving O_2_ transport at this higher point is considerably less than at sea level. It is obvious to consider that the PaO_2_ fall at each consecutive step in the O_2_ transport cascade is less at HAs than at sea level. Indeed, most of the humans have a great capacity for physiological adjustments to compensate for this reduced pressure gradient.

### 8.2. Pulmonary Ventilation

The most important feature of acclimatization is the increase in depth and rate of breathing, which results in an increase in alveolar ventilation that may reach 5-fold of the values at sea level [3, 9]. This is achieved by hypoxic ventilatory response (HVR) of the peripheral chemoreceptors, mainly the carotid bodies which are situated just above the bifurcation of the common carotid artery in response to the low O_2_ concentration in the arterial blood [4, 9]. The HVR is the reflex response to hypoxic stimulation of carotid body chemoreceptors. Ventilatory acclimatization to hypoxia includes the time-dependent increase in the HVR that occurs during hours to weeks of hypoxic exposure [3, 9]. Two major mechanisms have been described to explain the increase in the HVR during hypoxia [39]. First, the sensitivity of the carotid body glomus cells to O_2_ increases during chronic hypoxia. Second there is an increase in the CNS responsiveness to afferent input from the carotid body. Afferent fibers from the O_2_-sensing glomus cells of the carotid body reach to the brain via the carotid sinus nerve whose afferents project to the nucleus of the solitary tract. In turn, the nucleus of the solitary tract contains neurons that project to the phrenic motor nucleus. The phrenic nerve innervates the diaphragm and stimulates hyperventilation.

Hyperventilation increases partial pressure of alveolar (PPA) and PaO_2_ and decreases PPA and arterial CO_2_. In a study by West et al. [7], pulmonary gas exchange was studied on members of the American Medical Research Expedition to Everest at altitudes of 8,050 m, 8,400 m, and 8,848 m, respectively. Their results showed that the PPA of CO_2_ was reduced to 7 to 8 mm Hg, about one-fifth of its normal sea level value of 40 mm Hg. The alveolar PaO_2_ is then maintained near 35 mm Hg and arterial pH was 7.7 on the summit. Although some members of expedition had a much HVR to hypoxia at these extreme altitudes than others, there was approximately fivefold increase in the ventilatory rate when compared to resting levels.

Upon initial exposure to HAs the vital capacity and residual lung volume are reduced, but after about 4 weeks of residency, the values are maintained to a level that they are comparable to those measured at low altitudes [3, 37]. In a recent study, Sonmez et al. [40] measured vital capacity at different altitudes and the results showed that there was no statistically significant difference in vital capacity values after the measurements are taken at 1520 m, 3200 m, and 4200 m during one-week long climbing to Mountain Ararat (5138 m). The O_2_ pulmonary diffusing capacity remains unchanged at HAs when compared to the capacity attained at sea level [41]. 

## 9. Hematological Adaptations

Transport of O_2_ in the blood is mainly carried out by hemoglobin (Hb) which is present in RBCs. Upon initial exposure to HA, initial transient increase in erythrocyte concentration can be seen which is caused by a reduced plasma volume, not by an increased rate of erythrocyte production [3]. Tannheimer et al. investigated the influence of water distribution on Hb and hematocrit values during a long-term exposure at HA. Their results showed that the main reason for the observed rapid massive increase of Hb and hematocrit at altitude was an intravascular hemoconcentration effect provoked by a shift of fluid to the interstitium [42]. Reduced plasma volume is caused due to dehydration that is very common at HA, partly because of the great insensible fluid loss mainly caused by the large ventilation of cold dry air. A reduced fluid intake and probable diuresis may also be other factors causing initial plasma volume reduction. Krzywicki et al. [43] studied water metabolism during acute HA exposure during 6 days of HA (4,300 m). Their results showed that total body water was significantly decreased, extracellular water appeared to increase but not significantly, and intracellular water was significantly decreased at altitude. They concluded that with heavy physical activity prior to and during altitude exposure, it appeared that hypohydration and a diuresis still occurred during acute altitude exposure [43]. Over a course of a week in response to the hypoxia, the bone marrow is stimulated by erythropoietin to increase the production of RBCs. Erythropoietin (EPO) is a glycoprotein, which stimulates RBCs production. It is produced primarily in the kidney in response to hypoxia and/or endurance training. Athletes either live or sleep in artificial or natural hypoxic conditions with the aim to increase serum erythropoietin concentrations, which are thought to improve maximum oxygen uptake and thus exercise performance [44]. Erythropoiesis is central to optimizing performance at HA. During ascent to moderate or HAs, serum EPO levels typically peak within 24 to 48 h and then decline to near baseline levels within approximately one week [13, 45]. An increase in RBC mass is measurable after 3-4 weeks and further increases have been reported for up to 9 months of continuous altitude residence. For subjects who remain at HA for less than a week, the change in RBCs mass may not be considerable and would not make a significant contribution to the acclimatization process [46]. O_2_ concentration in the blood is also maintained with the changes in the affinity for O_2_. The affinity for O_2_ depends mainly on the acid-base status and the total concentration of organic phosphates in the erythrocyte, mainly 2,3-diphosphoglycerate (DPG) and ATP [46, 47]. DPG binds to Hb and decreases its affinity for O_2_ on exposure to HA. It was shown to increase slower and seems to reach a plateau only after the subjects spent several days at HA. While increased blood pH shifts the curve leftward reflecting increased affinity of Hb for O_2_, accumulation of RBC-DPG shifts the curve rightward reflecting decreased affinity for O_2_ [47]. It was found that RBC-DPG increased, but the predicted rightward shift was counterbalanced by an increasing blood pH with increasing altitude. O_2_ concentration remained relatively unchanged up to an altitude of 6300 m. Beyond this altitude, the curve progressively shifted leftward and the O_2_ concentration decreased because of the stronger effect of the high blood pH [48]. Relative increasing in capillary bed may lead to better blood perfusion and, thus, O_2_ could more readily diffuse despite the relatively low O_2_ concentration [3, 48]. Increases in skeletal muscle myoglobin levels have been reported following a relatively brief exposure to HA [49]. These increased levels may be another important factor for the availability of O_2_ in the tissues at HAs. These findings are compatible with the hypothesis that hypoxic training potentates skeletal muscle angiogenesis [49].

## 10. Metabolic Compensation

At HA, the cost of meeting tissue O_2_ requirement is competitive with other body functions that may become progressively impaired by alkalosis. The final situation represents a compromise between the respiratory stimuli, which is aimed at increasing blood alkalosis in order to optimize the O_2_ transport system and the metabolic adjustment, which is aimed at reestablishing normal blood pH. In other words, although hyperventilation is adaptive since it increases the arterial O_2_ levels, it is also nonadaptive because the hypoxia-induced decrease in PaCO_2_ at the alveolar level induces blood alkalization. Prolonged alkalosis, however, is not compatible with normal body homeostasis, as it impairs several functions, including those of the CNS [50]. Fortunately, the pH of the cerebrospinal fluid (CCF) changes towards normal by movement of bicarbonate out of the CCF, and the pH of the arterial blood moves towards normal by renal excretion of bicarbonate, after 2 or 3 days. By the help of this metabolic compensation, pH of the medullary chemoreceptors is lowered and the original relationship between the pH of the CCF and the blood is restored to sea level values. It is the maintenance of this equilibrium that enables the lowlanders to sustain increased ventilation at HAs without the risks of alkalosis or hypocapnia. The rate and extent of the metabolic compensation depend on the altitude being slower and less complete at very HAs [3, 50].

## 11. Cardiac Output (COP)

Altitude also affects COP, which is the other determinant of O_2_ delivery. Upon initial exposure to HA, the resting pulse rate increases rapidly from an average of 70 beats per minute to as much as 105 beats per minute in an attempt to compensate for the reduced O_2_ content of the blood [10, 51]. These changes are thought to be because of hypoxia-induced increase in sympathetic nerve activity and stimulation of beta adrenergic receptors of myocardium via both sympathetic fibers and circulating adrenaline resulting in abrupt augmentation of the COP [51, 52]. Another proposed mechanism for the increase in heart rate at HA is the partial parasympathetic withdrawal [53]. Underlying mechanism of the sympathetic overactivity unexplained and administration of O_2_ has been reported to have only minor effect on the elimination of chemoreflex activation [51, 53]. But increase in sympathetic nerve activity remains persistent even in well-acclimatized subjects [52]. With acclimatization, despite the increase in sympathetic nerve activity, heart rate and COP tend to fall [2]. This decline in COP appears to be associated with a decrease in heart rate which usually remains above sea level values and has been attributed to increased vagal input and to downregulation in the number of adrenergic receptors [2, 51, 52]. Another important component that leads to decrease in COP is the reduction in the stroke volume with acclimatization [9, 36]. Upon prolonged exposure to altitude, stroke volume clearly declines over time, stabilizing after 1-2 weeks. While the factors responsible for this alteration in stroke volume are unknown, hypoxic pulmonary artery vasoconstriction and loss of plasma volume which result in the reduction of preload may play a role in this decline [2, 36]. Despite the fall in COP discussed previously, the performance of the heart is well maintained even at extreme altitudes. There is no electrocardiographic evidence of myocardial ischemia, and cardiac contractility as assessed by ultrasound is well maintained despite the extreme conditions [12, 54]. During OE-II cardiac function was appropriate for the level of work performed and COP was not a limiting factor for performance.

## References

[1] Schoene RB (2008). Illnesses at high altitude. *Chest*.

[2] Palmer BF (2010). Physiology and pathophysiology with ascent to altitude. *The American Journal of the Medical Sciences*.

[3] West JB (2004). The physiologic basis of high-altitude diseases. *Annals of Internal Medicine*.

[4] Howald H, Hoppeler H (2003). Performing at extreme altitude: muscle cellular and subcellular adaptations. *European Journal of Applied Physiology*.

[5] Barry PW, Pollard AJ (2003). Altitude illness. *The British Medical Journal*.

[6] Bouchard C, Dionne FT, Simoneau JA, Boulay MR (1992). Genetics of aerobic and anaerobic performances. *Exercise and Sport Sciences Reviews*.

[7] West JB, Hackett PH, Maret KH, Milledge JS, Peters RM, Pizzo CJ (1983). Pulmonary gas exchange on the summit of Mount Everest. *Journal of Applied Physiology Respiratory Environmental and Exercise Physiology*.

[8] Mizuno M, Savard GK, Areskog NH, Lundby C, Saltin B (2008). Skeletal muscle adaptations to prolonged exposure to extreme altitude: a role of physical activity?. *High Altitude Medicine and Biology*.

[9] Calbet JAL, Lundby C (2009). Air to muscle O_2_ delivery during exercise at altitude. *High Altitude Medicine and Biology*.

[10] Naeije R (2010). Physiological adaptation of the cardiovascular system to high altitude. *Progress in Cardiovascular Diseases*.

[11] Green HJ, Sutton JR, Cymerman A, Young PM, Houston CS (1989). Operation Everest II: adaptations in human skeletal muscle. *Journal of Applied Physiology*.

[12] Hoppeler H, Vogt M (2001). Muscle tissue adaptations to hypoxia. *Journal of Experimental Biology*.

[13] Saunders PU, Pyne DB, Gore CJ (2009). Endurance training at altitude. *High Altitude Medicine and Biology*.

[14] Juel C, Lundby C, Sander M, Calbet JAL, van Hall G (2003). Human skeletal muscle and erythrocyte proteins involved in acid-base homeostasis: adaptations to chronic hypoxia. *Journal of Physiology*.

[15] Verges S, Bachasson D, Wuyam B (2010). Effect of acute hypoxia on respiratory muscle fatigue in healthy humans. *Respiratory Research*.

[16] Edwards LM, Murray AJ, Tyler DJ, Kemp GJ, Holloway CJ, Robbins PA (2010). The effect of high-altitude on human skeletal muscle energetics: P-MRS results from the Caudwell Xtreme Everest expedition. *PLoS One*.

[17] Westerterp KR, Kayser B (2006). Body mass regulation at altitude. *European Journal of Gastroenterology & Hepatology*.

[18] Rejc E, Lazzer S, Antonutto G (2010). Energy expenditure and dietary intake of athletes during an ultraendurance event developed by hiking, cycling and mountain climbing. *The Journal of Sports Medicine and Physical Fitness*.

[19] Ge RL, Wood H, Yang HH, Liu YN, Wang XJ, Babb T (2010). The body weight loss during acute exposure to high-altitude hypoxia in sea level residents. *Acta Physiologica Sinica*.

[20] Richalet JP, Robach P, Jarrot S (1999). Operation Everest III (COMEX '97): effects of prolonged and progressive hypoxia on humans during a smulated ascent to 8,848 m in a hypobaric chamber. *Advances in Experimental Medicine and Biology*.

[21] Macdonald JH, Oliver SJ, Hillyer K (2009). Body composition at high altitude: a randomized placebo-controlled trial of dietary carbohydrate supplementation. *The American Journal of Clinical Nutrition*.

[22] Sierra-Johnson J, Romero-Corral A, Somers VK, Johnson BD (2008). Effect of altitude on leptin levels, does it go up or down?. *Journal of Applied Physiology*.

[23] Kleessen B, Schroedl W, Stueck M, Richter A, Rieck O, Krueger M (2005). Microbial and immunological responses relative to high-altitude exposure in mountaineers. *Medicine and Science in Sports and Exercise*.

[24] MacDougall JD, Green HJ, Sutton JR (1991). Operation Everest II: structural adaptations in skeletal muscle in response to extreme simulated altitude. *Acta Physiologica Scandinavica*.

[25] Westerterp-Plantenga MS, Westerterp KR, Rubbens M, Verwegen CRT, Richelet JP, Gardette B (1999). Appetite at “high altitude” [Operation Everest III (Comex-'97)] a simulated ascent of Mount Everest. *Journal of Applied Physiology*.

[26] Dykiert D, Hall D, van Gemeren N (2010). The effects of high altitude on choice reaction time mean and intra-individual variability: results of the edinburgh altitude research expedition of 2008. *Neuropsychology*.

[27] Lowe M, Harris W, Kane RL, Banderet L, Levinson D, Reeves D (2007). Neuropsychological assessment in extreme environments. *Archives of Clinical Neuropsychology*.

[28] Sevre K, Bendz B, Hankø E (2001). Reduced autonomic activity during stepwise exposure to high altitude. *Acta Physiologica Scandinavica*.

[29] Lombardi C, Meriggi P, Agostoni P, Faini A, Bilo G, Revera M (2013). High-altitude hypoxia and periodic breathing during sleep: gender-related differences. *Journal of Sleep Research*.

[30] de Aquino Lemos V, Antunes HK, dos Santos RV, Lira FS, Tufik S, de Mello MT (2012). High altitude exposure impairs sleep patterns, mood, and cognitive functions. *Psychophysiology*.

[31] Weil JV (2004). Sleep at high altitude. *High Altitude Medicine and Biology*.

[32] Johnson PL, Edwards N, Burgess KR, Sullivan CE (2010). Sleep architecture changes during a trek from 1400 to 5000 m in the Nepal Himalaya. *Journal of Sleep Research*.

[33] Cingi C, Erkan AN, Rettinger G (2010). Ear, nose, and throat effects of high altitude. *European Archives of Oto-Rhino-Laryngology*.

[34] Latshang TD, Bloch KE, Lynm C, Livingston EH (2012). Traveling to high altitude when you have sleep apnea. *JAMA*.

[35] Anholm JD, Powles ACP, Downey R (1992). Operation Everest II: arterial oxygen saturation and sleep at extreme simulated altitude. *The American Review of Respiratory Disease*.

[36] Groves BM, Reeves JT, Sutton JR (1987). Operation Everest II: elevated high-altitude pulmonary resistance unresponsive to oxygen. *Journal of Applied Physiology*.

[37] Reite M, Jackson D, Cahoon RL, Weil JV (1975). Sleep physiology at high altitude. *Electroencephalography and Clinical Neurophysiology*.

[38] Nussbaumer-Ochsner Y, Ursprung J, Siebenmann C, Maggiorini M, Bloch KE (2012). Effect of short-term acclimatization to high altitude on sleep and nocturnal breathing. *Sleep*.

[39] Wolff CB (2000). Cerebral blood flow and oxygen delivery at high altitude. *High Altitude Medicine and Biology*.

[40] Sonmez GT, Ozen S, Yuktasir B, Yalcin B, Ozen G, Sonmez S (2009). The effects of high altitude climbing on respiratory parameters. *Medicina Sportiva*.

[41] Dill DB, Hillyard SD, Miller J (1980). Vital capacity, exercise performance, and blood gases at altitude as related to age. *Journal of Applied Physiology*.

[42] Tannheimer M, Fusch C, Böning D, Thomas A, Engelhardt M, Schmidt R (2010). Changes of hematocrit and hemoglobin concentration in the cold himalayan environment in dependence on total body fluid. *Sleep and Breathing*.

[43] Krzywicki HJ, Consolazio CF, Johnson HL, Nielsen WC, Barnhart RA (1971). Water metabolism in humans during acute high-altitude exposure (4,300 m). *Journal of applied physiology*.

[44] de P, Niebauer J (2012). Effects of high altitude training on exercise capacity: fact or myth. *Sleep and Breathing*.

[45] Sawka MN, Young AJ, Rock PB (1996). Altitude acclimatization and blood volume: effects of exogenous erythrocyte volume expansion. *Journal of Applied Physiology*.

[46] Basu M, Malhotra AS, Pal K (2007). Erythropoietin levels in lowlanders and high-altitude natives at 3450 m. *Aviation Space and Environmental Medicine*.

[47] Mairbäurl H, Oelz O, Bärtsch P (1993). Interactions between Hb, Mg, DPG, ATP, and Cl determine the change in Hb-O_2_ affinity at high altitude. *Journal of Applied Physiology*.

[48] Windsor JS, Rodway GW (2007). Research at the extremes: lessons from the 1981 American medical research expedition to Mt Everest. *Wilderness & Environmental Medicine*.

[49] Olfert IM, Breen EC, Mathieu-Costello O, Wagner PD (2001). Skeletal muscle capillarity and angiogenic mRNA levels after exercise training in normoxia and chronic hypoxia. *Journal of Applied Physiology*.

[50] Hornbein TF (2001). The high-altitude brain. *The Journal of Experimental Biology*.

[51] Mazzeo RS, Bender PR, Brooks GA, Butterfield GE, Groves BM, Sutton JR (1991). Arterial catecholamine responses during exercise with acute and chronic high altitude exposure. *American Journal of Physiology*.

[52] Hansen J, Sander M (2003). Sympathetic neural overactivity in healty humans after prolonged exposure to hypobaric hypoxia. *Journal of Physiology*.

[53] Hopkins SR, Bogaard HJ, Niizeki K, Yamaya Y, Ziegler MG, Wagner PD (2003). *β*-Adrenergic or parasympathetic inhibition, heart rate and cardic output during normoxic and acute hypoxic exercise in humans. *Journal of Physiology*.

[54] Windsor JS, Rodway GW, Montgomery HE (2010). A review of electrocardiography in the high altitude environment. *High Altitude Medicine and Biology*.

